# Off‐line effects of alpha‐frequency transcranial alternating current stimulation on a visuomotor learning task

**DOI:** 10.1002/brb3.1754

**Published:** 2020-07-27

**Authors:** Taiki Harada, Masayuki Hara, Kojiro Matsushita, Kenji Kawakami, Keisuke Kawakami, Masaya Anan, Hisato Sugata

**Affiliations:** ^1^ Department of Rehabilitation Oita University Hospital Oita Japan; ^2^ Graduate School of Science and Engineering Saitama University Saitama Japan; ^3^ Department of Mechanical Engineering Gifu University Gifu Japan; ^4^ Faculty of Welfare and Health Science Oita University Oita Japan; ^5^ Present address: Department of Rehabilitation Kagoshima University Hospital Kagoshima Japan

**Keywords:** aftereffect, alpha, EEG, off‐line condition, tACS, visuomotor learning task

## Abstract

**Introduction:**

It has been suggested that transcranial alternating current stimulation (tACS) at both alpha and beta frequencies promotes motor function as well as motor learning. However, limited information exists on the aftereffects of tACS on motor learning and neurophysiological profiles such as entrainment and neural plasticity in parallel. Therefore, in the present study, we examined the effect of tACS on motor learning and neurophysiological profiles using an off‐line tACS condition.

**Methods:**

Thirty‐three healthy participants were randomly assigned to 10 Hz, 20 Hz, or the sham group. Participants performed visuomotor learning tasks consisting of a baseline task (preadaptation task) and training task (adaptation task) to reach a target with a lever‐type controller. Electroencephalography was recorded from eight locations during the learning tasks. tACS was performed between the preadaptation task and adaptation task over the left primary motor cortex for 10 min at 1 mA.

**Results:**

As a result, 10 Hz tACS was shown to be effective for initial angular error correction in the visuomotor learning tasks. However, there were no significant differences in neural oscillatory activities among the three groups.

**Conclusion:**

These results suggest that initial motor learning can be facilitated even when 10 Hz tACS is applied under off‐line conditions. However, neurophysiological aftereffects were recently demonstrated to be induced by tACS at individual alpha frequencies rather than fixed alpha tACS, which suggests that the neurophysiological aftereffects by fixed frequency stimulation in the present study may have been insufficient to generate changes in oscillatory neural activity.

## Graphical abstract

Experimental setting and paradigm. A circular target was displayed randomly at any one of five locations that uniformly spanned a circle around the central starting point. Participants were instructed to control a lever‐type controller to reach the target with the cursor.

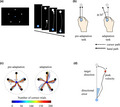

## 1 INTRODUCTION

In addition to the cerebellum, the primary motor cortex (M1) is a critical region for motor functions and motor learning (Galea, Vazquez, Pasricha, de Xivry, & Celnik, 2011; Hardwick, Rottschy, Miall, & Eickhoff, 2013; Nodera & Manto, 2014; Sugata et al., 2020; Wagner et al., 2019). Indeed, previous studies reported that M1 plays an important role in the stabilization of motor learning (Pollok, Latz, Krause, Butz, & Schnitzler, 2014; Baraduc, Lang, Rothwell, & Wolpert, 2004; Muellbacher, Ziemann, Boroojerdi, Cohen, & Hallett, 2001; Censor & Cohen, 2011; Sugata et al., 2014). In addition, the relationship between oscillatory neural activities generated from M1 and the ability for motor learning was recently reported (Pollok et al., 2014; Sugata et al., 2014; Yanagisawa et al., 2012). In particular, several studies demonstrated that neural profiles for low‐frequency components such as alpha and beta bands are associated with both motor function and motor learning (Pollok et al., 2014; Sugata et al., 2014; Yanagisawa et al., 2012). For example, alpha oscillation influences the visual and sensorimotor systems (Foxe, Simpson, & Ahlfors, 1998; Rihs, Michel, & Thut, 2007; Sugata et al., 2014; Yanagisawa et al., 2012; Zhuang et al., 1997) while beta oscillation facilitates motor function and motor learning (Boonstra, Daffertshofer, Breakspear, & Beek, 2007; Houweling, Daffertshofer, van Dijk, & Beek, 2008; Sugata et al., 2014). As such, the alpha band is considered to be related to visuomotor systems, while the beta band is associated with motor systems.

Transcranial alternating current stimulation (tACS) is a noninvasive brain stimulation technique that employs oscillatory electrical stimulation with the aim of facilitating neural activity at specific frequency bands (Tavakoli & Yun, 2017). Recent studies have shown that applied stimulation by tACS modulates neurophysiological and behavioral aspects in a frequency‐specific manner (Fröhlich, 2015; Herrmann, Rach, Neuling, & Strüber, 2013; Tavakoli & Yun, 2017). For example, researchers reported that motor function and motor learning can be modulated by 10 Hz and 20 Hz tACS over M1 (Pollok, Boysen, & Krause, 2015; Wach et al., 2013a). These findings suggest that tACS at alpha and beta frequencies over M1 has the possibility to modulate oscillatory neural activities and to improve motor function and motor learning. Accordingly, motor function and motor learning may be facilitated by changing oscillatory neural activities through externally applied alpha and beta tACS.

In the tACS method, two types of stimulation conditions were applied from the point of view of the task performance. These two types of simulations were termed “online” (Pollok et al., 2015) and “off‐line” conditions (Krause, Meier, Dinkelbach, & Pollok, 2016). In the online condition, tACS was applied “during” the task. On the other hand, in the off‐line condition, participants performed a given cognitive task first, then received tACS, and were then tested again on the same cognitive task without tACS. tACS. Many studies have focused on online condition because synchronized oscillatory neural activity at each frequency promotes neuronal plasticity (Pollok et al., 2015; Reato, Rahman, Bikson, & Parra, 2013; Wach et al., 2013b). In contrast, the effects of off‐line tACS have not been clarified, although several mechanisms were suggested (Reato et al., 2013). Recent studies reported that by using off‐line tACS, entrainment of oscillatory power changes by tACS lasted more than 30 min (Wach et al., 2013a; Wach et al., 2013b; Kasten, Dowsett, & Herrmann, 2016), and an improvement in motor function was observed after alpha and beta tACS (Krause et al., 2016; Wach et al., 2013a). Furthermore, the aftereffects of tACS by alpha and beta frequencies were reported to modulate both behavioral and physiological profiles in parallel (Kasten et al., 2016). However, the aftereffects of tACS at alpha and beta frequencies on behavioral and neurophysiological profiles related to motor learning remain to be clarified.

Considering the findings that behavioral and neurophysiological profiles can be modulated by alpha and beta tACS, we hypothesized that performance of motor learning and oscillatory neural activities could be equally modulated after alpha and beta tACS. To test this hypothesis, we examined the aftereffects of tACS on motor leaning and related oscillatory neural activities using visuomotor learning tasks and electroencephalography (EEG) in human participants. These series of results suggest that it is useful for studying neurorehabilitation for diseases such as stroke that require motor learning.

## 2 MATERIAL AND METHODS

### 2.1 Participants

Thirty‐three healthy volunteers (age: 21.82 ± 5.73, male/female: 12/21) participated in this study. All participants were right‐handed, which was confirmed using the Edinburgh Handedness Inventory Test (Oldfield, 1971). All participants presented with normal or corrected‐to‐normal vision. General exclusion criteria included a history or family history of epileptic seizures, brain‐related injury, other neurological or psychiatric disorders, and pregnancy. All participants were naive with respect to the precise purpose of the study and never received transcranial electrical stimulation before. In accordance with the Declaration of Helsinki, we explained the purpose and possible consequences of this study to all participants and obtained their informed consent before the study commenced. This study was conducted with the approval of the Oita University Medical School Ethical Review Boards (Approved number 1184‐T1).

### 2.2 Experimental setup and paradigm

Using a between‐subjects design, participants were randomly assigned to 10 Hz tACS (*n* = 11), 20 Hz tACS (*n* = 11), or the sham (*n* = 11) group. All participants were blinded to the stimulation parameters. Detailed information on each stimulation group is shown in Table 1.

**Table 1:** Characteristics of participants in experimental groups

	10 Hz tACS	20 Hz tACS	Sham tACS	*p*‐Value
Age	21.4 ± 4.6	22.5 ± 8.5	21.6 ± 3.3	.904
Gender [males (females)]	5 (6)	3(8)	4(7)	.697
Handedness (%)	98.9 ± 3.5	100 ± 0	91.2 ± 25.0	.439
				Mean ± *SD*
				*p* < .05

Note

One‐way analysis of variance indicated no significant differences among the groups.

### 2.3 Visuomotor learning tasks

**Figure 1:**
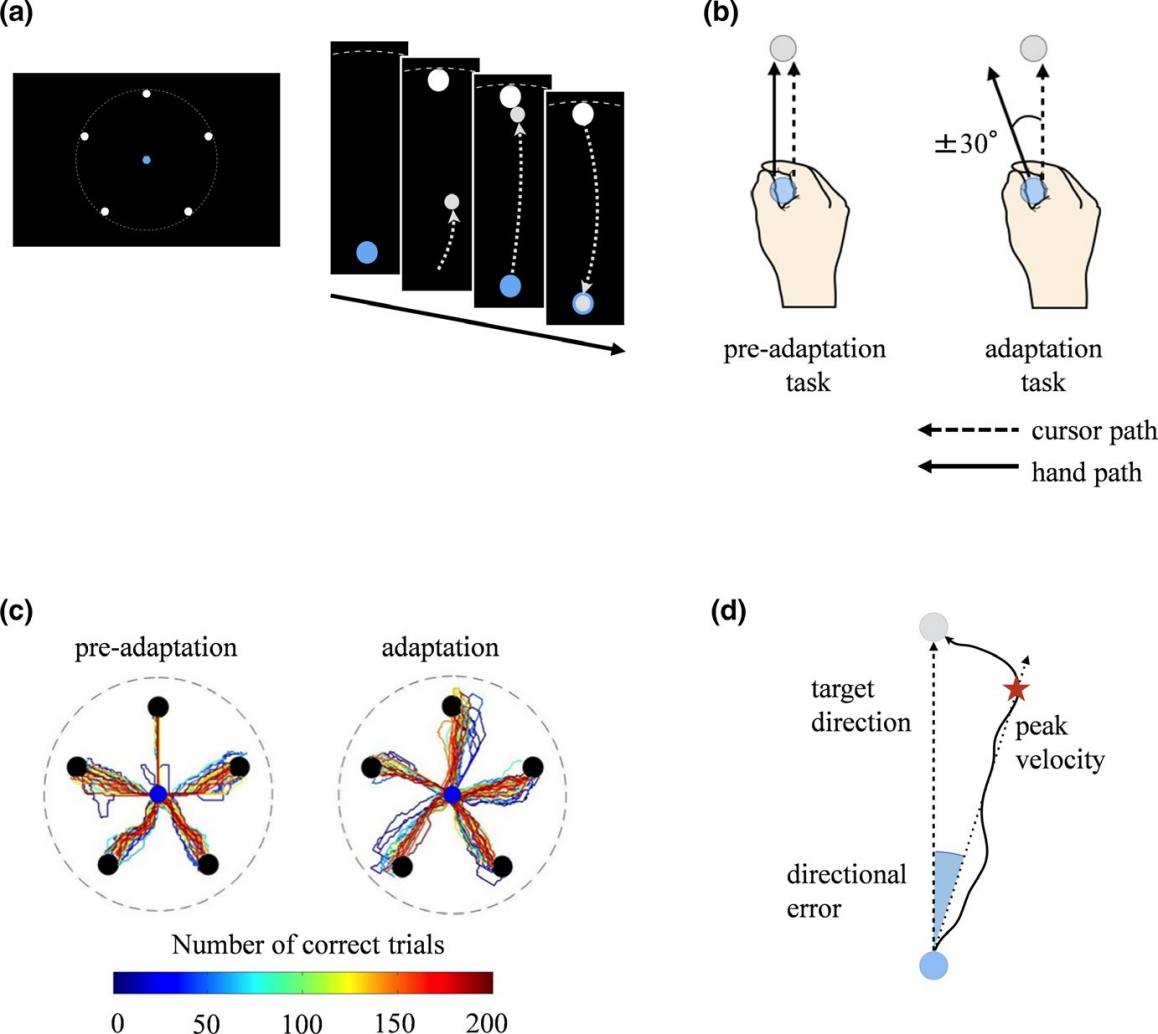
Experimental setting and paradigm. (a) A circular target was displayed randomly at any one of five locations that uniformly spanned a circle around the central starting point. Participants were instructed to control a lever‐type controller to reach the target with the cursor. (b) In the preadaptation task, the direction of the cursor movement was the same as that of the hand movement. In the adaptation task, the direction of the cursor movement was rotated 30° clockwise or counterclockwise from the direction of the hand movement. The dot line indicates the cursor path, while the solid line indicates the hand path. (c) Representative data in the preadaptation task and adaptation task. In the adaptation task, the hand path was gradually corrected as participants learned the directional error. (d) The angle between the direct line from the start position to target position (dashed line) and the line representing the direction movement at the peak outward velocity (dotted line) was calculated and defined as the directional error

To examine whether tACS facilitates motor learning, a visuomotor learning task based on a previous study (Huber, Ghilardi, Massimini, & Tononi, 2004) was applied. In the present study, the following two types of center‐out reaching tasks were performed before and after tACS; a baseline task (preadaptation task; before tACS) and a training task (adaptation task; after tACS). In each task, subjects sat in front of a desk. There was a monitor displaying the target, and the movement of the cursor and a lever‐type controller (Extreme 3D Pro Joystick; Logicool Co Ltd.) was provided on the desk. A circle target was displayed randomly at any one of five locations that uniformly spanned a circle of around the central starting point. The participants kept the pointer in the central starting point for 2,000 ms. The participants were instructed to move the pointer in time with the beeping sound which was the onset cue. On hearing the cue, the subject moved the pointer toward the target as quickly as possible and within the shortest possible distance within 2,500 ms (Figure 1a). In the preadaptation task, the direction of the cursor movement was the same as that of the hand movement. In the adaptation task, unbeknown to the participants, the direction of the cursor movement was rotated 30° clockwise or counterclockwise from the direction of the hand movement (Figure 1b). Representative example data from the preadaptation task and adaptation task are shown in Figure 1c. The angular transformation was counterbalanced in each group.

### 2.4 EEG measurements

EEG measurements were recorded using active electrodes (Polymate Mini AP108; Miyuki Giken Co., Ltd). Eight electrodes (F3, F4, C3, Cz, C4, P3, Pz, and P4) were placed according to the International 10–20 system, and the electrode impedance did not exceed 20 kΩ. EEG electrodes were composed of a sintered Ag and AgCl material. The ground electrode was located on the forehead, and the reference was mounted on the left earlobe. The active electrode was attached directly on the scalp with an EEG conductive paste.

**Figure 2:**
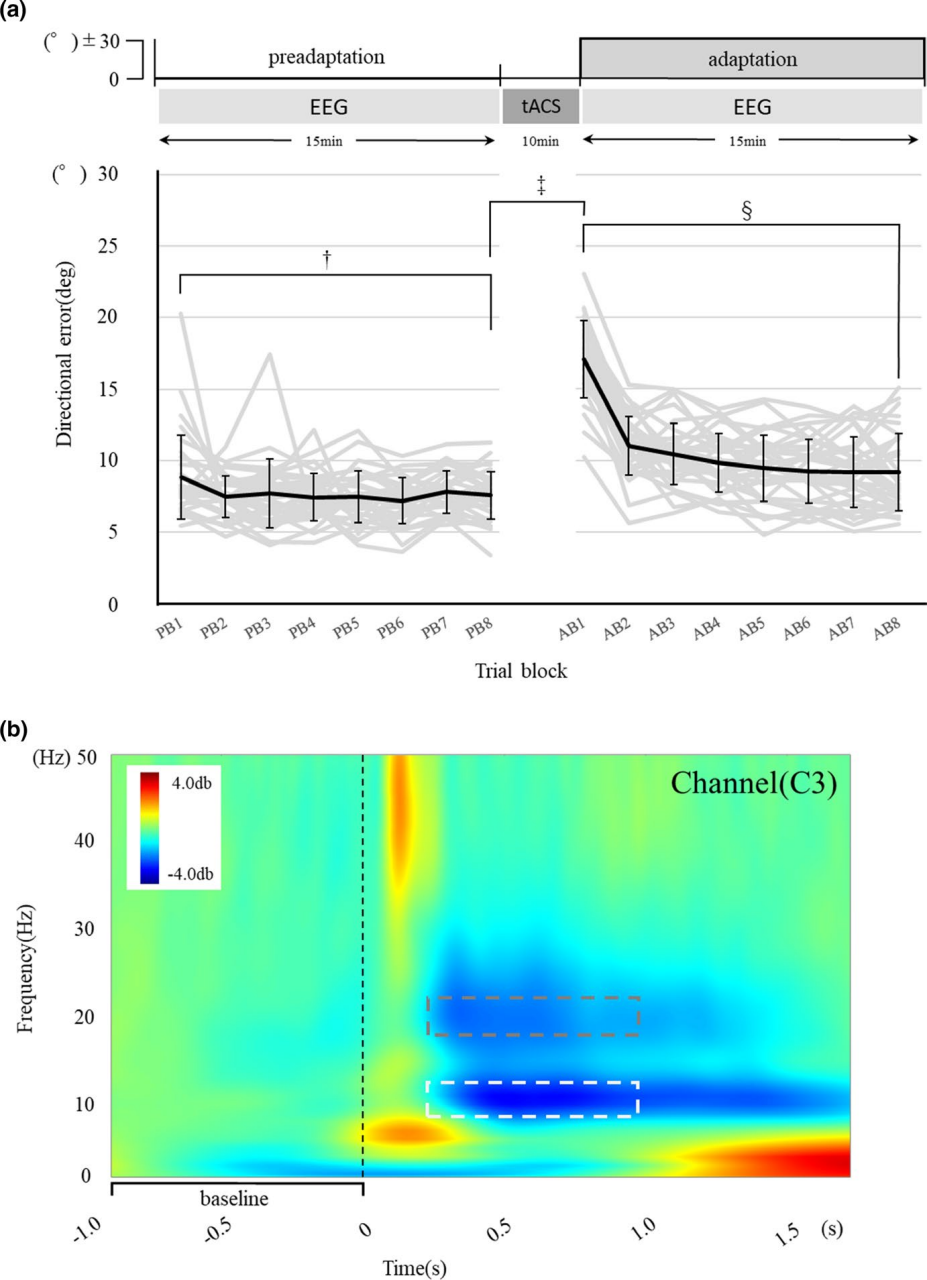
(a) The time course of the visuomotor learning task and the EEG measurement is shown. Directional errors were compared following three patterns among the three groups; † (Δ PB8‐PB1); ‡ (ΔAB1‐PB8); and § (Δ AB8‐AB1). TACS was applied between the preadaptation task and the adaptation task. (b) Grand averaged time–frequency plot in the adaptation tasks for all participants. Representative C3 electrode data are shown. The average values of the event‐related neural oscillatory activities in the alpha (white box) and beta bans (gray box), based on the 250‐ to 1,000‐ms poststimulus window, were compared before and after tACS. The black dashed line represents the onset cue of the movement

EEG data were sampled at a rate of 500Hz. To reduce the contamination of eye movement artifacts, participants were instructed to fix their eyes on the display without unnecessary eye movement. EEG was recorded for 15 min during each task. In other words, no EEG was recorded during the 10 min while receiving tACS at each frequency (Figure 2a). Therefore, the active electrode (C3) was removed before attaching the sponge electrode, and the electrode was reattached after stimulation. Saline solution that was not necessary was removed before attaching the active electrode. If necessary, the active electrode was fixed with paste after polishing with a pretreatment agent. The measurement was started after confirming that the impedance was 20 Ω or less.

### 2.5 tACS sessions

The participants received tACS between the preadaptation and adaptation tasks, which was delivered with a battery‐driven constant current stimulator (DC stimulator plus, NeuroConn, Ilmenau, DEU) through a pair of saline‐soaked sponge electrodes (5 × 7 cm). Participants were seated in a comfortable chair with their eyes closed during the tACS or sham stimulation. The electrode positions were decided in accordance with the International 10–20 system. According to previous studies (Antal et al., 2008; Pollok et al., 2015; Sugata et al., 2018), the target electrode was located on C3, and the other electrode placed over the right orbital. After removing the EEG C3 electrodes, tACS electrodes were fixated using a Velcro band. Stimulation was applied for 10 min with 1 mA (peak‐to‐peak amplitude). Impedance values were maintained below 10 kΩ. The setup for the sham stimulation group was the same, with the exception that no current passed through the electrode. Among all participants, 7 in 10 Hz tACS and 9 in 20 Hz tACS recognized the phosphenes. No other side effects were perceived and all participants underwent stimulation.

### 2.6 Behavioral analyses

First, in order to calculate the directional error angle, the peak velocity for the hand path from the initial hand position to the position of the target was calculated for each trial. Second, the directional error was extracted by determining the angle between the direct line from the start to target position and the line representing the direction of movement at the peak outward velocity (Figure 1d) (Huber et al., 2004).

We defined a series of five different target trials as one epoch. Within each epoch, the order of the five target trials was randomized and the entire task consisted of 40 epochs (200 trials). Epochs were divided into 8 blocks, and the peak velocity and angular error were averaged for each block in the preadaptation task (preadaptation block1‐8: PB1‐PB8) and adaptation task (adaptation block1‐8: AB1‐AB8), respectively.

### 2.7 EEG data analyses

EEG data were analyzed using Brainstorm software (Tadel, Baillet, Mosher, Pantazis, & Leahy, 2011). An off‐line band‐pass filter between 0. 1 and 100 Hz with a notch at 60 Hz was performed to eliminate environmental noise. Electromyography‐contaminated trials were visually detected and manually eliminated. The beep sounds indicating go cue were defined as 0 ms. The time window of an epoch was defined as −1,500 to 2,500 ms. Therefore, one trial in the EEG analysis was regarded as one trial of visuomotor learning task analysis. A total of 200 trials were analyzed and divided into 8 blocks as well as tasks. For examining the effect of tACS on oscillatory neural activities, time–frequency analysis was applied to active electrodes for determining the oscillatory neural activities, as shown in previous studies (Pollok et al., 2014; Sugata et al., 2018). Event‐related oscillatory neural activities were calculated as two‐dimensional (latency by frequency) representations of the mean change in spectral power (in dB) relative to baseline, which ranged from −1,000 to 0 ms before the onset of the stimulus. The period of interest ranged from 0 to 2,000 ms after stimulus onset. Each epoch was subjected to short‐time Fourier analysis using the fast Fourier transform. Then, averaged power of oscillatory neural activity was calculated in the alpha band (9–11 Hz) and beta band (19–21 Hz) based on a 250–1,000 ms post‐stimulus window (Figure 2b). These frequency bands were extracted to demonstrate the effect of tACS on oscillatory neural activity. Thus, each frequency band was defined as tACS stimulation frequency ± 1 Hz.

### 2.8 Statistical analyses

Statistical analyses were performed using SPSS (version 25) and MATLAB (R2017a). First, in order to show whether the participants in each group had similar motor skills, differences in the directional error and peak velocity between PB1 and PB8 were compared among the stimulation groups by one‐way analysis of variance (ANOVA). Next, to examine the effect of tACS on immediate changes in motor function, differences in the directional error and peak velocity between PB8 and AB1 were compared among the stimulation groups. Finally, differences in the directional error and peak velocity between AB1 and AB8 were compared among the stimulation groups to confirm the effect of tACS on the improvement of visuomotor learning (Figure 2a) (Krause et al., 2016; Pollok et al., 2015; Sugata et al., 2018). Furthermore, oscillatory neural activities before and after tACS were examined from eight EEG channels to demonstrate the effect of tACS. As shown in a previous study (Antal et al., 2008), three‐way ANOVA of *time*, *channel*, and *stimulation* was applied to the EEG data. The Bonferroni correction was used for post hoc analysis in ANOVA. The significance level for all statistical tests was set to *p* < .05.

## 3 RESULTS

### 3.1 Directional error in the preadaptation task and adaptation task

**Figure 3:**
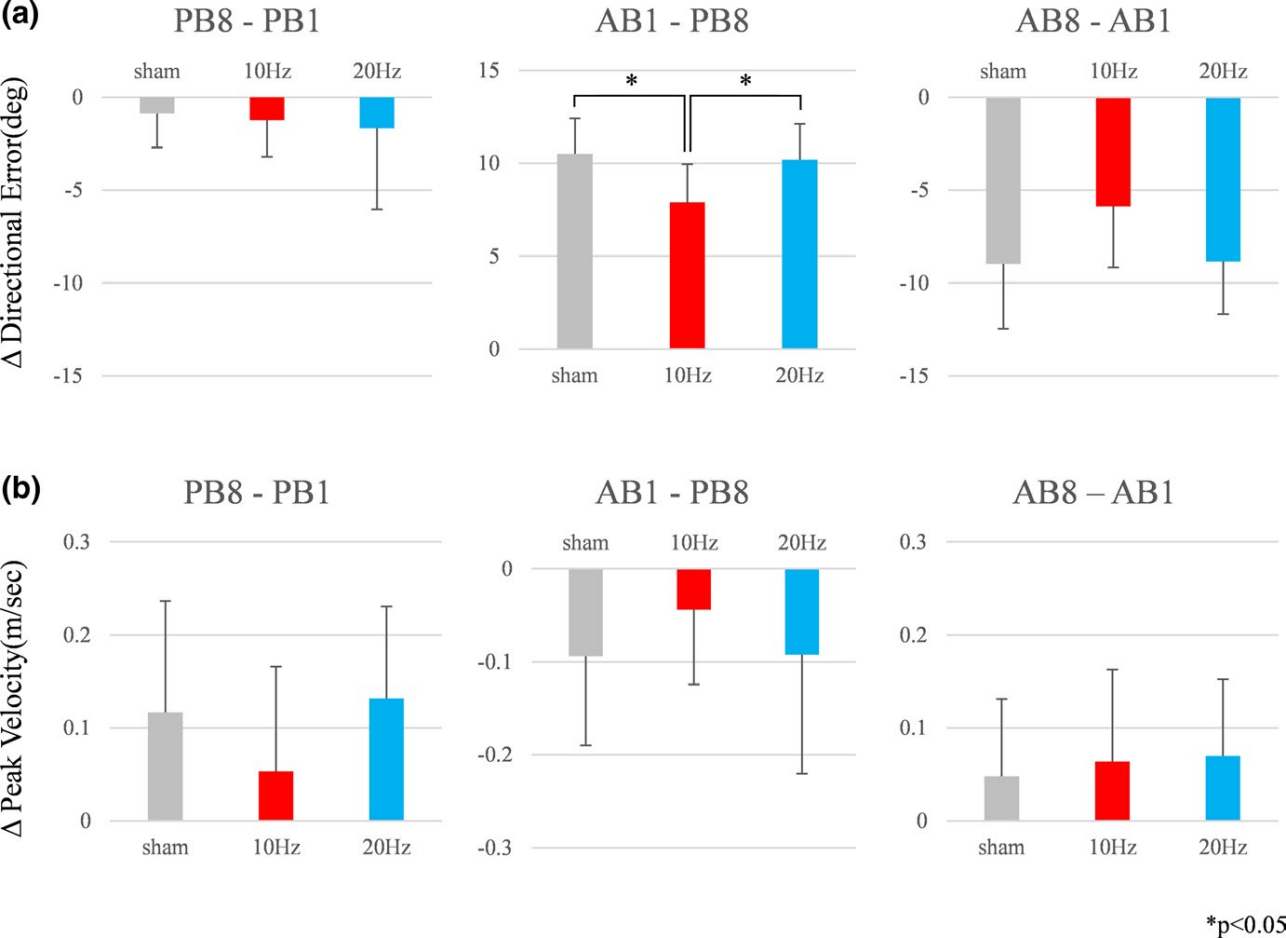
(a) Δ directional error in each block. Error bars indicate standard deviations (*SD*). A significant difference was obtained between AB1 and BP8 among the three groups (*p* < .01). Multiple comparison with Bonferroni correction showed the significant differences between 10 and 20 Hz (*p* < .05) and between 10Hz and sham (*p* < .05). (b) Differences in peak velocity each block showed no significant differences among the three groups. Error bars indicate *SD*

We first examined changes in the directional error before and after tACS. Our results showed significant differences between PB8 and AB1 among the three groups (*F*_2,30_ = 5.756, *p* = .008, *η^2^p* = 0.277). In order to further investigate these differences, a multiple comparison with Bonferroni correction was performed. Our results demonstrated significant differences between the 10 and 20 Hz groups (*p* = .031) and between the 10 Hz and sham groups (*p* = .012) but not between the 20 Hz and sham groups (*p* = 1.000) (Figure 3a middle). No significant differences in directional error between PB1 and PB8 (*F*_2,30_ = 0.199, *p* = .821, *η^2^p* = 0.013) (Figure 3a left) and AB1 and AB8 (*F*_2,30_ = 3.265, *p* = .052, *η^2^p* = 0.179) were observed among the three groups (Figure 3a right).

### 3.2 Peak velocity in the preadaptation task and adaptation task

Peak velocity was compared among the three groups before and after tACS. Differences in the peak velocity between PB1 and PB8 (*F*_2,30_ = 1.544, *p* = .230, *η^2^p* = 0.024) (Figure 3b left), PB8 and AB1 (*F*_2,30_ = 0.829, *p* = .446, *η^2^p* = 0.018) (Figure 3b middle), and AB1 and AB8 (*F*_2,30_ = 0.167, *p* = .847, *η^2^p* = 0.016) showed no significant differences (Figure 3b right).

### 3.3 Oscillatory neural activities before and after tACS

In order to examine the potential relationship between oscillatory neural activity and behavioral aspects after tACS, oscillatory neural activities were calculated in eight EEG channels and compared before and after tACS.

In the alpha band, three‐way ANOVA with the factors *time* (PB8 vs. AB1), *stimulation* (10 Hz vs. 20 Hz vs. sham), and *channels* (F3, F4, C3, Cz, C4, P3, Pz, and P4) revealed a significant main effect of *time* (*F*_1,30_ = 3.385, *p* = .035, *η^2^p* = 0.014) but not *stimulation* (*F*_2,30_ = 0.067, *p* = .977, *η^2^p* = 0.0001) and *channels* (*F*_7,30_ = 1.212, *p* = .295, *η^2^p* = 0.018), *stimulation × time* (*F*_2,30_ = 2.351, *p* = .072, *η^2^p* = 0.015), *stimulation × channel* (*F*_14,30_ = 0.341, *p* = .991, *η^2^p* = 0.011), *channel × time* (*F*_7,30_ = 0.573, *p* = .778, *η^2^p* = 0.008), and *stimulation × time × channel* interactions (*F*_14,30_ = 0.073, *p* = 1.000, *η^2^p* = 0.002) (Table 2).

**Table 2:** The result of three‐way ANOVA of EEG

	*df*	*F* value	*p*‐Value	*η* ^2^ *p*
Alpha band				
*Stim*	2	0.067	.977	0.0001
*Time*	1	3.385	.035*	0.014
*Channel*	7	1.212	.295	0.018
*Stim* × *Time*	2	2.351	.072	0.015
*Stim* × *Channel*	14	0.341	.991	0.011
*Channel* × *Time*	7	0.573	.778	0.008
*Stim* × *Channel* × *Time*	14	0.073	1.000	0.002
Beta band				
*Stim*	2	3.995	.019*	0.016
*Time*	1	0.243	.622	0.001
*Channel*	7	3.261	.002*	0.045
*Stim* × *Time*	2	2.104	.123	0.009
*Stim* × *Channel*	14	0.184	1.000	0.005
*Channel* × *Time*	7	0.382	.913	0.006
*Stim* × *Channel* × *Time*	14	0.072	1.000	0.002

Note

Independent variables: preadaptation block (PB8) and adaptation block (AB1) (*Time*), condition of current stimulation (10 Hz, 20 Hz, sham) (*Stim*), and EEG channels (eight channels: F3, F4, C3, Cz, C4, P3, Pz, P4) (*Channel*); dependent variable: FFT power in a given frequency band. The three‐way ANOVA revealed no significant interactions between current conditions, time, and channels at any of the different frequencies applied. The asterisk indicates significant *p*‐values (*p* < .05).

* *p* < .05

In the beta band, three‐way ANOVA with the factors *time* (PB8 vs. AB1), *stimulation* (10 Hz vs. 20 Hz vs. sham), and *channels* (F3, F4, C3, Cz, C4, P3, Pz, P4) revealed a significant main effect of *stimulation* (*F*_2,30_ = 3.995, *p* = .019, *η^2^p* = 0.016) and *channels* (*F*_7,30_ = 3.261, *p* = .002, *η^2^p* = 0.045) but not *stimulation × time* (*F*_2,30_ = 2.104, *p* = .123, *η^2^p* = 0.009), *stimulation × channel* (*F*_14,30_ = 0.184, *p* = 1.000, *η^2^p* = 0.005), *channel × time* (*F*_7,30_ = 0.382, *p* = .913, *η^2^p* = 0.006) and *stimulation × time × channel* interactions (*F*_14,30_ = 0.072, *p* = 1.000, *η^2^p* = 0.002) (Table 2).

## 4 DISCUSSION

In the present study, initial angular error correction in PB8‐AB1 during the visuomotor learning tasks was significantly facilitated after 10 Hz tACS compared with that after sham and 20 Hz tACS. Conversely, there were no significant differences in angular error correction among the three groups at the AB8. Furthermore, there were no significant differences in peak velocity among the three groups in each block.

Recent studies demonstrated the potential for 10 Hz stimulation to enhance motor performance under both online (Pollok et al., 2015) and off‐line (Wach et al., 2013a) conditions. For instance, Pollok et al reported that 10 Hz tACS facilitates the retrieval of newly learned motor performance (Pollok et al., 2015), and Antal et al. (2008) reported that 10 Hz tACS facilitates sequential motor learning. Conversely, researchers reported that 20 Hz tACS facilitates motor function and stabilizes learned motor performance under online (Pollok et al., 2015) and off‐line (Krause et al., 2016) conditions. Twenty Hz tACS was also reported to slow down movement velocity (Pogosyan, Gaynor, Eusebio, & Brown, 2009; Wach et al., 2013a). Collectively, these results suggest that 10 and 20 Hz tACS facilitate motor performance, while 20 Hz tACS additionally stabilizes motor function during both online and off‐line conditions. In addition to the above studies, several aftereffects of 10 Hz tACS with regard to behavioral aspects were recently reported (Krause et al., 2016; Wach et al., 2013a). For example, sustained behavioral changes were reported at 30 min after 10 Hz tACS (Wach et al., 2013a). Furthermore, Fresnoza et al reported that tACS at individual alpha frequencies (IAF) induces robust behavioral aftereffects (Fresnoza et al., 2018).

As for oscillatory neural profiles, several studies demonstrated the neural profiles for low‐frequency components such as alpha and beta bands (Klostermann et al., 2007; Neuper, Wörtz, & Pfurtscheller, 2006; Rektor, Sochůrková, & Bocková, 2006), and they showed that these frequencies exhibit a functional diversity in cortico‐basal networks that are simultaneously activated during sensorimotor processing (Klostermann et al., 2007). For example, alpha oscillation has been shown to influence both visual and sensorimotor systems (Foxe et al., 1998; Rihs et al., 2007; Sugata et al., 2014; Yanagisawa et al., 2012; Zhuang et al., 1997), while beta oscillation facilitates motor function and motor learning (Boonstra et al., 2007; Houweling et al., 2008; Sugata et al., 2014). In the present study, 10 Hz tACS, but not 20 Hz tACS, was effective for initial angular error correction. Considering that the alpha band is related to visuomotor function, the initial angular error correction observed in the present study may reflect the aftereffect of 10 Hz tACS on motor learning in the initial stage of the visuomotor learning tasks.

However, in this study, there was no significant difference in angular error correction between the three groups in AB8, whereas previous studies reported aftereffects of alpha tACS on motor learning (Krause et al., 2016; Wach et al., 2013a). In particular, tACS at IAF has been reported to induce robust behavioral aftereffects (Fresnoza et al., 2018). Since the present study applied tACS at fixed frequencies such as 10 and 20 Hz, but not individual frequency, we may not have obtained the robust aftereffect of tACS on motor learning in AB8. Furthermore, the visuomotor learning tasks used in the present study would be considered higher‐order motor learning, which requires proprioceptive and visual feedback to control movements, a process more relevant to the development and learning of a new sport or a musical instrument (Manuel, Guggisberg, Thézé, Turri, & Schnider, 2018). This suggests that the aftereffect of tACS at fixed frequency, that is 10 Hz, may not be sufficient to further correct the angular error or overcome the ceiling effect in the visuomotor learning tasks. Thus, the angular error correction immediately after tACS may reflect the facilitation of initial motor learning by 10 Hz tACS. These results support the notion that initial motor learning can be facilitated even when 10 Hz tACS is applied under off‐line conditions.

In contrast, there were no significant differences in angular error correction and peak velocity among three groups in 20 Hz tACS. Twenty Hz tACS was reported to facilitate motor function, stabilize learned motor performance (Krause et al., 2016; Pollok et al., 2015), and slow down movement velocity (Pogosyan et al., 2009; Wach et al., 2013a). Furthermore, Sugata et al. (2018) reported that motor learning capacity was modulated at 10 Hz tACS compared with 20 Hz tACS. Considering these reports, 10 Hz tACS may facilitate motor learning more than 20 Hz tACS. Therefore, there may not be significant difference in 20 Hz tACS.

In the present study, we also investigated the aftereffects of tACS on oscillatory neural activities after tACS. However, there were no significant differences in oscillatory neural activities before and after tACS among the three groups.

Recently, researchers suggested that online tACS effects are associated with the entrainment of neural oscillation, whereas off‐line tACS effects are related to plastic changes such as the long‐term‐potentiation driven changes of synaptic weight (Herrmann et al., 2013; Veniero, Vossen, Gross, & Thut, 2015; Vossen, Gross, & Thut, 2015; Zaehle, Rach, & Herrmann, 2010). Accordingly, the aftereffects of tACS are likely due to synaptic plasticity, not to entrainment. In fact, many studies recently reported the aftereffects of off‐line tACS on neurophysiological profiles (Antal et al., 2008; Krause et al., 2016; Sugata et al., 2018; Wach et al., 2013b). Indeed, the aftereffect of alpha tACS at IAF has been demonstrated to persist for at least 30 min in the alpha band (Kasten et al., 2016). In addition, persistent cortical excitability was reported after tACS at IAF (Fresnoza et al., 2018). However, while some studies reported robust aftereffects of alpha tACS on oscillatory neural activities (Kasten et al., 2016; Neuling, Rach, & Herrmann, 2013; Zaehle et al., 2010), several studies reported weak alpha‐tACS aftereffects (Antal et al., 2008; Fekete, Nikolaev, De Knijf, Zharikova, & van Leeuwen, 2018; Stecher & Herrmann, 2018). For instance, Helfrich et al reported that power changes of alpha frequency after 10 Hz tACS persisted for only 1 min (Helfrich et al., 2014), and Antal et al. (2008) reported behavioral effects during 10 Hz tACS but no neurophysiological effects after 10 Hz tACS. Furthermore, an animal study using tACS reported no neurophysiological aftereffects (Reato et al., 2013). Collectively, these results imply that aftereffects of alpha tACS on neurophysiological profiles may be induced by IAF stimulation rather than fixed 10 Hz stimulation. In the present study, we applied not individual alpha‐ and beta‐band frequencies but fixed stimulation frequencies, such as 10 and 20 Hz, and no significant differences in oscillatory neural activity were observed among the three groups. Given that robust neurophysiological aftereffects are induced by tACS at IAF rather than fixed alpha tACS (Fresnoza et al., 2018; Reato et al., 2013; Rektor et al., 2006), neurophysiological aftereffects by fixed frequency stimulation in the present study may have been insufficient to generate changes in oscillatory neural activity. Thus, the no significant changes in oscillatory neural activities before and after tACS observed in the present study may be due to the stimulation frequency in the alpha range. In other words, tACS at IAF may result in more robust behavioral and neurophysiological changes and may show further motor learning effects. Considering that oscillatory neural activity in the alpha band tended to be modulated by 10 Hz tACS (*p* = .072 (stimulation × time)), the neurophysiological aftereffects of alpha tACS on visuomotor learning may be clarified by increasing the number of participants even when fixed 10 Hz frequency is applied to stimulation.

In contrast, several studies have focused on the effect of tACS in patients with neurological disease. For example, stimulating chronic stroke with online beta tACS improved the classification accuracy of the neurofeedback interventions compared with before stimulation (Naros & Gharabaghi, 2017). Furthermore, 20 Hz tACS attenuated beta band cortico‐muscular coupling during isometric contraction and amplitude variability during finger tapping in patients with Parkinson's disease (Krause et al., 2014). However, 10 Hz tACS had no effect. These differences may be due to the differences in task or stimulation parameters and disease specificity. Thus, 10 Hz tACS can be effective for patients who need visuomotor learning such as those experiencing stroke. These series of results suggest that it is useful in the study of neurorehabilitation for diseases such as stroke that require motor learning.

The present study has several limitations. First, although a previous study showing the aftereffects of tACS on oscillatory neural activity and motor function used multichannel MEG (Sugata et al., 2018; Wach et al., 2013b), we recorded from only eight EEG channels. Therefore, we cannot rule out the possibility that tACS‐induced oscillatory neural activity could not be detected due to its low spatial resolution. In addition, the sample size was small. A sample size of more than 20 subjects has been recently recommended (Cohen, 2017). Thus, the statistical power of the present study may be weak. The neurophysiological aftereffects of 10 Hz tACS on visuomotor learning may be clarified by increasing the number of participants. Second, a recent study reported that tACS effects were caused not by direct transcranial stimulation to cortical neurons but by synchronized cortical activities induced by percutaneous stimulation of the peripheral nerves of the skin (Asamoah, Khatoun, & Mc Laughlin, 2019). However, we did not evaluate changes in the peripheral nerves after tACS. Thus, we cannot speculate on the effects of tACS on oscillatory neural activity via percutaneous stimulation of the peripheral nerves of the skin. Third, electromyography‐contaminated trails were visually detected and manually eliminated; however, vertical electrooculography was not performed in this study. Thus, we could not rule out the possibility that eye‐blink artifacts contaminated the EEG results. To further address these problems, further research is warranted.

## 5 CONCLUSIONS

In this study, we investigated the effect of off‐line 10 and 20 Hz tACS on visuomotor learning tasks. Angular error correction was significantly facilitated immediately after 10 Hz tACS. However, there were no significant differences in oscillatory neural activities before and after tACS in each group. This result suggests the initial motor learning can be facilitated by 10 Hz tACS even when 10 Hz tACS is applied under off‐line conditions. Conversely, neurophysiological aftereffects by fixed frequency stimulation in the present study might have been insufficient to generate changes in oscillatory neural activity.

## CONFLICT OF INTEREST

None of the authors declare any conflict of interest.

## AUTHOR CONTRIBUTIONS

Taiki Harada contributed to methodology, investigation, writing of original draft, and visualization. Masayuki Hara contributed to software provision and methodology. Kojiro Matsushita performed formal analysis. Kenji Kawakami contributed to investigation and provision of resources. Keisuke Kawakami contributed to investigation and provision of resources. Masaya Anan performed formal analysis. Hisato Sugata contributed to conceptualization, methodology, investigation, software provision, validation, formal analysis, data curation, writing of original draft, review and editing, visualization, project administration, and funding acquisition.

## Data Availability

The data that support the findings of this study are available from the corresponding author upon reasonable request.
